# The Politics of Global Public Health in Fragile States and Ungoverned Territories

**DOI:** 10.1371/currents.dis.ba3beede71ca0746a0972aa3837ed618

**Published:** 2017-01-09

**Authors:** Frederick M. Burkle

**Affiliations:** Harvard Humanitarian Initiative, Harvard University, Cambridge, Massachusetts, USA; The Woodrow Wilson International Center for Scholars, Washington, DC, USA

## Abstract

The reasons for global health crises and how the world responds to them have dramatically changed over the last half century. Increasingly, natural disasters result in failure of public health and security systems leading to preventable conflict, unconventional war and unprecedented population migration. While scientific expertise exists to mitigate these failures in fragile states and ungoverned territories, inactions are mired by the lack of political will, international legal mandates, and capacity to strategically monitor multidisciplinary public health indicator failures.

## Article

In 2004 I was honored to be interviewed for the Lancet medical journal’s Lifeline Series.^1^ I had just come away from a disastrous short tenure as the Interim Minister of Health in Iraq following the 2003 war. I had support from former Secretary of State Colin Powell to rapidly mitigate and recover the war related destruction of essential public health infrastructure and protections required as Occupiers under Articles 55 and 56 of the Geneva Convention (GC) that follow every war. Predictably, the loss of essential public health protections in food, water, sanitation, shelter, health, and energy leads to excess and preventable mortality and morbidity, numbers that exceed those from war weaponry by 50-70% or more.^2^ This plan was immediately squelched by an unprecedented decision within the Bush Administration that moved these post-hostilities humanitarian responsibilities from the State Department to the Department of Defense under Donald Rumsfeld. This decision claimed that US forces were not ‘occupiers’ but ‘liberators’, promptly reversing any previously planned public health recovery and rehabilitation. The State Department’s coterie of seasoned nation-building experts, including myself, was summarily replaced. Before leaving Iraq I publicly declared Baghdad a public health emergency but this too fell on operational deaf ears.^3^ Many in Iraq see that decision as the most egregious of policies enacted after the invasion, in which the elderly, children, woman and disabled primarily suffered the most. While the ‘liberator’ claim was debunked and reversed 18 months later, it was too late. Without a reliable public health data and surveillance system, also thwarted in the war’s aftermath, the political powers remained protected from further scrutiny. Thirteen years later, Iraq remains a public health emergency.^4^

The *Lifeline* interview asked several questions: “What did I believe was the most exciting field of science?” My answer: “Public health. It has the most potential and the least support.” The interviewer, surprised, stated that to date no one had ever mentioned public health. When asked what I thought was the greatest political danger to the medical profession I answered: “Political interference in public health.”^1^

My answer today would be the same. We continue to see how the decades old international legal framework is easily overwhelmed by political inaction, interference and moreover, struggles for relevance given today’s modern challenges. The reasons for humanitarian crises and how the world must respond to them have dramatically changed every decade.^5^ The 1945 United Nations (UN) Charter, International Humanitarian Law (IHL) and the 1949 GC was designed to protect humanitarian aid in cross border wars. Unfortunately, while the language remains relevant and attempts have been made to adapt to modern day intrastate wars, too few warring factions and signatory governments either respect, or are unwilling, incapable, or selectively and blatantly ignore the protections we in the humanitarian community found sacred. No longer is their assurance for the continued safety of citizens, military casualties ’out of combat’, vital public health infrastructure and protections, and humanitarian personnel in intrastate unconventional armed conflicts. As political oppression and armed conflict erupts, essential public health infrastructure rapidly disappears and populations flee. If one accepts that disasters keep us honest by defining the public health and exposing its vulnerabilities, the global community must emphasize prevention and preparedness and re-legitimize it under international law to ensure protective strategies that intercede in fragile states before they deteriorate to the point of no return.

More than a decade after the Iraq war, a broader brand of global health engagement has emerged yet public health’s role within that rubric remains in limbo, is operationally ignored, or is ill defined. What sanctioned interventions exist ‘under international law’ to protect the public health before conditions deteriorate? None are clearly defined. Working from existing laws of war, the ICRC, influenced by the consequences of Iraq and now Syria, acknowledge the overwhelming and dramatic “cumulative impact that stems from the complexity of urban system” collapse and their mutual dependence on country-wide large-scale inter-connected infrastructure loss that the health systems are not able to keep up with.^6^ The numbers killed or injured are unprecedented. While today we painstakingly attempt to document the loss of health personnel in war, there is no equal documentation of essential public health recovery personnel, especially in water and sanitation.^6^ Despite the desperate call for a renewed emphasis on disaster risk reduction in 2015’s Hyogo Framework for Action, the fledgling global community is fixed on interventions that still favor response over preparedness and prevention for natural disasters. But what if the consequences of a natural disaster, including that of climate change, are inextricably leading to conflict or war?

Today’s domestic and regional crises are increasingly under the influence of widely integrated global changes and forces defined by climate change, biodiversity loss, emergencies of water, food and energy scarcity and rapid unsustainable urbanization. These crises, initially slow moving, are increasingly severe affecting massive populations across many borders. Drought, crop destruction, and famine coincide with loss of vital aquifers. Whatever limited and often primitive public health protections remain, they have proved ineffectual, dangerously managed and selectively denied to the most vulnerable by those in power who persistently ignore wide ranging mitigation advances offered by the scientific community.

New legal preventive protocols and epidemiologic surveillance approaches are needed to protect civilians. Protecting the public health must be viewed both as a strategic and security issue requiring close collaboration with humanitarian, and military logistical and security personnel. Any attempt to redefine public health as a security issue must be coupled with efforts to develop a more comprehensive accounting of the human cost of modern-day fragile and ungoverned territories—not just warfare.

A mandate for a universally accepted system of preventive monitoring of more precise methods and outcome indicators that measure the effectiveness and efficiency of national health and public health systems is undeniable. However, health alone cannot solve these global health problems. While some standard indicators are already available, the most sensitive are often multi-and trans-disciplinary. For example, rates of dengue fever, which escalate when trash collection is inadequate, are sensitive indicators for economists of both poor governance and urban decay.^7^ The humanitarian community is far from realizing this goal. For instance, we do not know how to operate effectively in unsustained dense rapidly urbanized settlements, a most likely site of future major conflicts. Unless measures are taken to develop ways to include indirect mortality and morbidity, calculating the human cost of public health decline will remain an inexact process of estimation by political scientists, humanitarians and military analysts. Capacity to access vital information of the location, function and extent of destroyed essential infrastructure is currently “not accessible.”^8^ The lives at risk and those lost will remain unseen, uncounted and unnoticed—and the lessons for effective prevention and protections unlearned.

Crises only gain international attention when they result in conflict. The Syrian conflict is a case in point. From 2011 to 2016, 60% of Syria’s agricultural northeast and south suffered its worst drought, water shortage and crop failure, compounded by failures in governance and management. Poverty accelerated the exodus of farmers, herders and rural families to cities in the west fomenting today’s major sectarian war. Multiple public health interventions were available and could have ceased or mitigated the decline and population exodus.^9^ Similar lost opportunities for preventive engagement occurred in the Sudan, Somalia, Eritrea and Ethiopia.

Indeed, if all the forcibly displaced persons would be placed in one state it would be the 21st largest populated country in the world.^10^ Populations escaping from public health collapse---as internally displaced or refugees---will exceed those from warfare alone, further adversely affecting the fragile public health protections in host countries such as Jordan, Lebanon, Turkey, and Greece. The humanitarian community, strongly adhering to the global political commitment of ‘responsibility to protect’ endorsed by all member states of the UN must also recognize that migrants have an equal right to live and thrive in the country and culture in which they were raised.^11^ Not surprisingly, many migrants to the EU have openly declared their dream to return to their native country.

UN sanctioned revisions and rewrites of the IHL and the GC are crucial. The ICRC reports that there is “still room to strengthen and clarify the existing legal framework” to “adapt to new realities”; and, talks of supplemented GC Commentaries that “will give state and non-state actors an understanding of the law as it is widely interpreted today so that it is widely applied effectively in modern armed conflicts.”^12^

More than ever, we need strong international humanitarian laws and an effective accountability and recourse mandate for those who fail to respect the laws that are in place. Why wait for conflicts to occur when we have a clear evidence-based global mandate to mitigate the obvious public health consequences? With public health infrastructure and protections “absent, destroyed, overwhelmed, not recovered or maintained, or denied to populations” it has become a massive global health emergency.^13^

While we have had ‘laws of war’ for centuries, is it time, in an increasingly globalized world plagued by public health emergencies, for laws of prevention? Public health protections are a human right. What can one hopefully say to an emerging global society’s credibility that it has the tools to wage war but not to prevent them? The scientific expertise exists to be a force in preparedness and prevention; the political will and international law mandates must follow.

## Competing Interests

The opinions expressed in this article are the author's own and do not reflect the view of their affiliated institutions.

## Corresponding Author

Frederick M. Burkle, Jr.: skipmd77@aol.com
